# A Rare Case with Systemic Lupus Erythematosus Manifested by two Different Neurologic Entities; Guillain Barre Syndrome and Posterior Reversible Encephalopathy Syndrome

**DOI:** 10.31138/mjr.31.3.358

**Published:** 2020-09-30

**Authors:** Firdevs Ulutaş, Veli Çobankara, Uğur Karasu, Nuri Baser, Ismail Hakkı Akbudak

**Affiliations:** 1 Department of Rheumatology; 2 Department of Internal Medicine; 3 Department of Intensive Care Unit, Faculty of Medicine, Pamukkale University, Denizli, Turkey

**Keywords:** Systemic lupus erythematosus, Guillain-Barre Syndrome, posterior reversible encephalopathy syndrome

## Abstract

Systemic lupus erythematosus (SLE) is an immune-mediated, lifelong disease characterized by quite heterogeneous neuropsychiatric manifestations. Herewith, we report the first rare co-incidental case with posterior reversible encephalopathy syndrome (PRES), Guillain Barre Syndrome (GBS), and (SLE). The coexistence of these neurological conditions in SLE patients could lead to delayed diagnosis and treatment due to this rare coalescence and clinical diversity. Currently, there are no specific, diagnostic radiological or laboratory biomarkers for neurological involvement in SLE. Awareness and, early recognition of neuropsychiatric involvements of the disease are important for timely appropriate treatment. Delayed treatment may cause permanent damage, poor prognosis, long term morbidity, and even death.

## INTRODUCTION

Systemic lupus erythematosus (SLE) is an immune-mediated, lifelong disease. Vasculitis of small vessels, deposition of immune complexes, and autoantibody production and deposition in various organs play a role in the disease pathogenesis. Neuropsychiatric manifestations of SLE, including headaches, seizures, psychosis, and delirium, are quite heterogeneous and may occur at the onset of lupus or later in the course of the disease. The frequency of neurologic involvement ranges between 12%–95% in SLE patients.^1^ Peripheral nervous system involvement is in less than 10% of all nervous system manifestations.^2^ The simultaneous prevalence of SLE with Guillain Barre Syndrome (GBS) has been reported to be between 0.6% and 1.7%, whereas the prevalence of posterior reversible encephalopathy syndrome (PRES) is 0.69% in SLE.^3^ The coexistence of PRES and GBS in SLE patients has not been reported in the past. Prompt diagnosis and treatment of patients can be delayed due to this rare coalescence and clinical diversity. Also, many confusing clinical conditions including hypertensive and uremic encephalopathy, delirium, use of cytotoxic drugs, ischemic or haemorrhagic cerebrovascular events, and infections may have added to the neurological involvement.^4^ Although it is difficult to find overlapping effects of predisposing factors in these patients, making the distinction of the above clinical conditions is very important for treatment modalities. In the literature, this is the first educational case where GBS and PRES occurred together in an SLE patient.

## CASE REPORT

**Figure 1:**
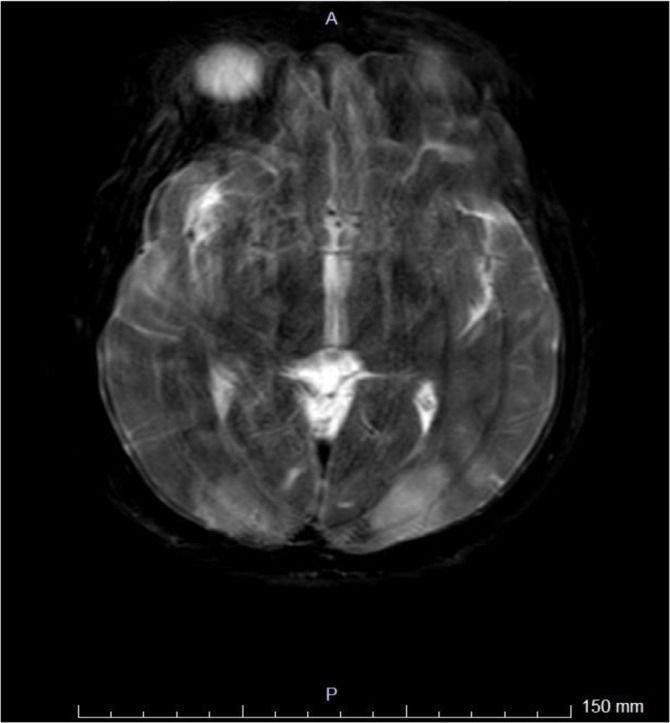
T2 weighted image of brain MRI 39-year-old man with systemic lupus erythematosus, presenting with headache and seizures after cytotoxic treatment and hypertension. Hyperintense lesions, posterior subcortical right side predominant vasogenic white matter edema is seen indicating PRES.

**Figure 2:**
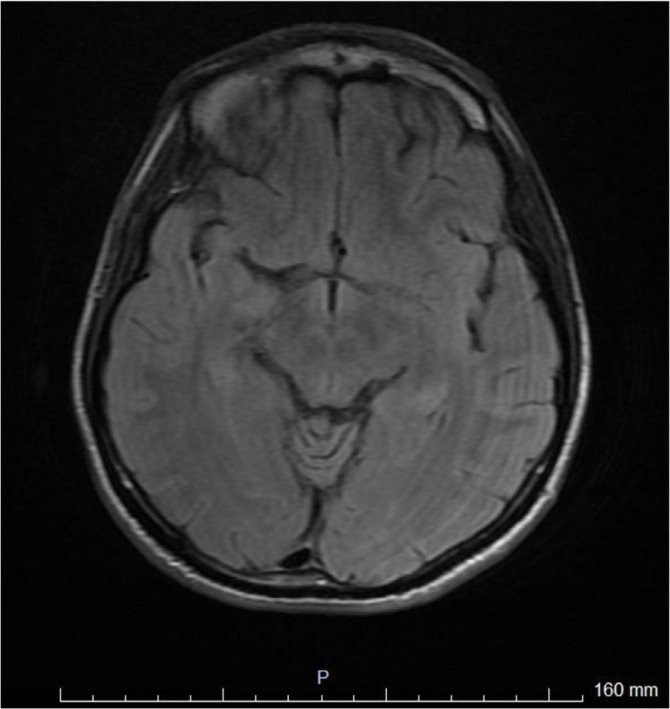
FLAIR MRI; complete resolution of vasogenic edema after supportive treatments

A 39-year-old man with a previous history of urogenital infection one year ago, was admitted to the inpatient clinic of Neurology at Pamukkale University, Denizli. He complained of acute onset lower extremity pain, numbness and progressive weakness, stiffness in the ankles, and walking instability for the previous two weeks. At presentation, he was afebrile and normotensive. He was unable to stand alone. He had no malar rash, no oral ulcers, no weight loss, no alcohol or illicit drug use, and no high-risk sexual behaviour. On physical exam, the muscle strength in his legs was 3/5 proximally and 2/5 distally and his deep tendon reflexes were absent. The pattern in the distal limbs resembled acute onset symmetric ascending paraparesis. Bilateral axillary and inguinal 1–2 cm superficial lymphadenopathies were noted. Cerebrospinal fluid and nerve conduction studies showed albuminocytologic dissociation and findings of dysautonomic inflammatory demyelinating polyneuropathy, respectively. He was firstly treated with standard therapies including intravenous immunoglobulin (IVIG) and plasmapheresis sessions for the diagnosis of Guillain Barre Syndrome, but did not respond well to these therapies. Brain and whole spine magnetic resonance imaging was normal and inguinal excisional lymph node biopsy resulted in reactive lymphoid hyperplasia. Serum angiotensin converting enzyme (ACE) level was normal, and no lymph node or parenchymal involvement was detected in lung tomography. After excluding sarcoidosis, infections, malignancies, and antiphospholipid antibody syndrome, he was diagnosed as SLE with the presence of symmetrical arthralgia and positive immunological markers including positive antinuclear antibody, anti-dsDNA antibody, anti-Smith antibody, and anti-SSA antibody with low complement levels, meeting the minimum 4 of 11 classification criteria created by the American College of Rheumatology. Laboratory tests revealed no hematological or renal involvement. Intravenous pulse methylprednisolone (1000 mg per day, 5 consecutive days) and cyclophosphamide (1000 mg single dose per month) were given. After intensive immunosuppressive treatments, he had tonic clonic seizures and severe headaches. Hypertension was observed (160/110). Concurrent infections and cerebrovascular insults were excluded. His brain magnetic resonance imaging (MRI) was consistent with hyperintense white matter vasogenic edema, predominantly the right side of the posterior brain on T2 weighted images (**Figure 1**). He was treated with antiepileptic and antiedema therapies. Parenteral antihypertensive agents were titrated for adequate blood pressure control. Post-treatment, complete resolution of vasogenic edema was observed on fluid-attenuated inversion recovery (FLAIR) MRI images (**Figure 2**). Thus the diagnosis of Posterior Reversible Encephalopathy Syndrome (PRES) was made. Despite aggressive intensive care management, no clinical improvement was seen and the patient subsequently died as a result of the neurological insult.

## DISCUSSION

This is the first original educational case where GBS and PRES occurred together in an SLE patient. Only 15 cases with PRES and GBS have been reported in the recent literature, with the vast majority of patients being female and older than the age of 55.^5^ PRES can develop in association with autoimmune diseases like SLE, GBS, and polyarteritis nodosa. Although the association between PRES and GBS are poorly understood, underlying possible mechanisms leading to PRES in GBS patients may include dysautonomia, autoimmunity, IVIG therapy, and activation of the sympathetic nervous system. Dysautonomic cardiac and cerebrovascular complications of GBS include tachy-bradycardia, hypo-hypertension, and PRES, respectively.^6^ The rare coexistence of PRES and GBS has also been reported after spinal surgery,^7^ in association with hyponatremia and IVIG therapy^8^ and head injury.^9^

The 75% of patients with SLE can present with many different neuropsychiatric manifestations from headache to stroke throughout the course of the disease.^10^ In patients with GBS, autoantibodies target peripheral nervous system cells. As a result, damaged nerves cannot transmit signals from the brain to the muscles. Many studies showed that antecedent viral or bacterial infectious agents like Campylobacter play a role in the etiology and may trigger autoimmune peripheral neuropathy. The recent cases of GBS and SLE in the literature were treated successfully with a combination of IVIG, corticosteroids, plasma exchange, and/or intravenous cyclophosphamide (CyC). The response rate of treatments was stated as 77.4% of patients with different types of peripheral nervous system involvement.^11^ Although multiple clinical trials demonstrated the significant benefits of intravenous immunoglobulin and plasma exchange for the treatment of GBS, our patient did not respond to initial treatment modalities and his clinical condition worsened.^12^ During the intensive immunosuppressive treatment including CyC and pulse steroid, he developed posterior reversible encephalopathy syndrome (PRES). This was first described in 1996 by Hinchey.^13^ This neuroradiological disease is characterized by classical symptoms like headache, altered mental function, visual symptoms, vomiting, seizures, and with typical bilateral posterior subcortical brain edema on magnetic resonance imaging. PRES is completely reversible with supportive treatments and does not require immunosuppressive drugs. The rate in SLE patients was 18% in a case series of 120 patients with PRES and patients diagnosed with SLE and PRES were analyzed retrospectively. Concurrent hypertension, treatment with high dose steroids, and CyC have also been reported as risk factors.^14^ CyC is a mainstay drug for neurolupus and lupus nephritis and may trigger PRES via direct endothelial cytotoxic effects at the blood brain barrier.^15^ In another study, renal insufficiency and high SLE Disease Activity Index (SLEDAI) were also shown to be risk factors for the development of PRES.^16^ Cui Hw et al. stated that SLE patients with PRES had more early disease onset with predominantly seizures, and higher mortality rates than controls.^17^ Severe hypertension disrupts the autoregulation of brain blood flow. Also, interleukin-6 (IL-6)-related inflammation and endothelial damage are thought to cause hyperperfusion induced vasogenic edema and brain injury in SLE, which show a high mortality rate.^18^ Male gender, atypic presentation with GBS, early disease onset, and unresponsiveness to previous treatment modalities were poor prognostic factors for our patient.

Herewith, we report the first rare coincidental case with PRES, GBS, and SLE. Underlying possible conditions in this patient were not clear. The underlying autoimmune diseases including SLE and GBS, concurrent hypertension, the use of cyclophosphamide (CyC), and IVIG may be predisposing causes for the development of PRES. We did not have a chance for further distinction due to the lethal outcome of the disease. Nevertheless, we surmise that cyclophosphamide-related PRES developed rather than active lupus disease. The patient already had been treated with intensive immunosuppressive drugs for active disease.

## CONCLUSIONS

Currently, there are no specific, diagnostic radiological or laboratory biomarkers for neurological involvement in SLE. Awareness and early recognition of neuropsychiatric involvements of the disease are important for timely and appropriate treatment. Delayed treatment may cause permanent damage, poor prognosis, long term morbidity, and even death. We hope that this case could raise awareness of atypical presentations of neuropsychiatric involvement in SLE patients.

## References

[1] Kampylafka E Alexopoulos H Kosmidis M Panagiotakos DB Vlachoyiannopoulos PG Dalakas MC Incidence and prevalence of major central nervous system involvement in systemic lupus erythematosus: a 3-year prospective study of 370 patients. PLoS ONE 2013;8:e55843. 23424638 10.1371/journal.pone.0055843 PMC3570560

[2] Hanly JG Urowitz MB Sanchez-Guerrero J Bae SC Gordon C Wallace DJ Neuropsychiatric events at the time of diagnosis of systemic lupus erythematosus: an international inception cohort study. Arthritis Rheum 2007;56:265–73. 17195230 10.1002/art.22305

[3] Nadri Q Althaf MM Guillian-Barre syndrome as the initial presentation of systemic lupus erythematosus-case report and review of literature. Ann Saudi Med 2015;35:263–5. 26409804 10.5144/0256-4947.2015.263 PMC6074455

[4] Tatjana Zekic Benic Mirjana Stanic Antulov Ronald Antončić I Novak S The multifactorial origin of posterior reversible encephalopathy syndrome in cyclophosphamide-treated lupus patients. Rheumatol Int 2017;37:2105–14. 29043491 10.1007/s00296-017-3843-x

[5] Chen A Kim J Henderson G Berkowitz A Posterior Reversible Encephalopathy Syndrome in Guillain-Barre Syndrome. J Clin Neurosci 2015;22:914–6. 25800144 10.1016/j.jocn.2014.11.004

[6] Lovie J Igbokwe E Hinchey J Posterior Reversible Encephalopathy Syndrome associated with the Dysautonomia of Guillain-Barre Syndrome. Neurol Bull 2009;1:7–10.

[7] Sanpei Y Hanazono A Kamada S Sugawara M Guillain Barre Syndrome and Posterior Reversible Encephalopathy Syndrome following Spinal Surgery. Case Rep Neurol 2019;11(3):284–9. 31607895 10.1159/000502570 PMC6787417

[8] Drye C Bose S Pathireddy S Aeddula NR Guillain-Barre syndrome with concurrent posterior reversible encephalopathy syndrome and hyponatremia: mere coincidence. BMJ Case Rep 2019;12(7). pii: e229749. 10.1136/bcr-2019-229749 PMC6626439 31300598

[9] Yonekura S Anno T Kobayashi N Posterior Reversible Encephalopathy Syndrome and Guillain-Barre syndrome after Head Injury: Case Report. Neurol Med Chir (Tokyo) 2018;58(10):453–8. 30078819 10.2176/nmc.cr.2018-0049 PMC6186763

[10] Kakati S Barman B Ahmed SU Hussain M Neurological manifestations in systemic lupus erythematosus: a single centre study from North East India. J Clin Diagn Res 2017;11:OC05–OC09. 10.7860/JCDR/2017/23773.9280 PMC5324435 28273990

[11] Van Doorn P Diagnosis, treatment and prognosis of Guillain-Barre syndrome (GBS). La Presse Medicale 2013;42:e193–e201. 23628447 10.1016/j.lpm.2013.02.328

[12] Toledano P Orueta R Rodriguez-Pinto I Valls-Solé J Cervera R Espinosa G Peripheral nervous system involvement in systemic lupus erythematosus: prevalence, clinical and immunological characteristics, treatment and outcome of a large cohort from a single centre. Autoimmun Rev 2017;16:750–5. 28483540 10.1016/j.autrev.2017.05.011

[13] Chevret S Hughes RA Annane D Cochrane Neuromuscular Group Plasma Exchange, Intravenous immunoglobulin for Guillain Barre syndrome. Cochrane Database Syst Rev 2017;(2):CD001798.28241090

[14] Fugate JE Claassen DO Cloft HJ Kallmes DF Kozak OS Rabinstein AA Posterior reversible encephalopathy syndrome: associated clinical and radiologic findings. Mayo Clin Proc 2010;85:427–32. 20435835 10.4065/mcp.2009.0590 PMC2861971

[15] Houssiau FA Vasconcelos C D’Cruz D Sebastiani GD Garrido Ed Ede R Danieli MG Immunosuppressive therapy in lupus nephritis: the Euro-Lupus Nephritis Trial, a randomized trial of low-dose versus high-dose intravenous cyclophosphamide. Arthritis Rheum 2002;46:2121–31. 12209517 10.1002/art.10461

[16] Jung SM Moon SJ Kwok SK Ju JH Park KS Park SH Posterior reversible encephalopathy syndrome in Korean patients with systemic lupus erythematosus: risk factors and clinical outcome. Lupus 2013;22:885–91. 23846231 10.1177/0961203313496341

[17] Cui Hw Lei RY Zhang SG Han LS Zhang BA Clinical features, outcomes and risk factors for posterior reversible encephalopathy syndrome in systemic lupus erythematosus: a case-control study. Lupus 2019;28(8):961–9. 31208267 10.1177/0961203319856416

[18] Fragoso-Loyo H Richaud-Patin Y Orozco-Narvaez A Dávila-Maldonado L Atisha Y Llorente L Interleukin-6 and chemokines in the neuropsychiatric manifestations of systemic lupus erythematosus. Arthritis Rheum 2007;56:1242–50. 17393453 10.1002/art.22451

